# Levels of sex steroid hormones and their receptors in women with preeclampsia

**DOI:** 10.1186/s12958-020-0569-5

**Published:** 2020-02-18

**Authors:** Kuo-Chung Lan, Yun-Ju Lai, Hsin-Hsin Cheng, Ni-Chin Tsai, Yu-Ting Su, Ching-Chang Tsai, Te-Yao Hsu

**Affiliations:** 1 Department of Obstetrics and Gynecology, Kaohsiung Chang Gung Memorial Hospital and Chang Gung University College of Medicine, 123 Ta-Pei Road, Niao-Sung District, Kaohsiung City, Kaohsiung Taiwan; 2 Center for Menopause and Reproductive Medicine Research, Kaohsiung Chang Gung Memorial Hospital and Chang Gung University College of Medicine, Kaohsiung, Taiwan; 3 Graduate Institute of Clinical Medicine, College of Medicine, Kaohsiung Medical University, Kaohsiung, Taiwan

**Keywords:** Preeclampsia, Placenta, Sex steroids, Steroid receptor

## Abstract

**Background:**

Pregnant women have high serum concentrations of sex steroid hormones, which are major regulators of paracrine and autocrine responses for many maternal and placental functions. The main purpose of this study was to compare patients with preeclampsia and patients with uncomplicated pregnancies in terms of serum steroid hormones (estradiol [E2], progesterone [P4], dehydroepiandrosterone sulfate [DHEAS], and testosterone [T]) throughout pregnancy and the levels of cord blood and placental steroid receptors during the third trimester.

**Methods:**

Quantitative real-time reverse transcription PCR, western blotting, and immunohistochemistry were used to determine the levels of steroid hormones in the serum and cord blood and the placental levels of estrogen receptor-α (ERα), ERβ, androgen receptor (AR), and progesterone receptor (PR).

**Results:**

There were 45 women in the uncomplicated pregnancy group and 30 women in the preeclampsia group. Serum levels of T were greater and serum levels of E2 were reduced in the preeclampsia group, but the two groups had similar levels of P4 and DHEAS during the third trimester. Cord blood had a decreased level of DHEAS in the preeclampsia group, but the two groups had similar levels of P4, E2, and T. The two groups had similar placental mRNA levels of ERα, ERβ, AR, and PR, but the preeclampsia group had a higher level of ERβ protein and a lower level of ERα protein. Immunohistochemistry indicated that the preeclampsia group had a greater level of ERβ in the nucleus and cytoplasm of syncytiotrophoblasts and stromal cells.

**Conclusions:**

Women with preeclampsia had lower levels of steroid hormones, estrogen, and ERα but higher levels of T and ERβ. These molecules may have roles in the pathogenesis of preeclampsia.

## Background

Preeclampsia is a pregnancy-specific hypertensive disorder with multisystem involvement that is associated with an increased risk of future cardiovascular disease [1]. The pathogenesis of preeclampsia has not been fully elucidated, but much progress has been made in recent decades [2]. In particular, it is widely accepted that preeclampsia is associated with abnormal placentation, reduced placental perfusion, and systemic vasospasm. A two-stage model of preeclampsia proposed that incomplete spiral artery remodeling in the uterus contributes to placental ischemia and the release of antiangiogenic factors from the ischemic placenta into the maternal circulation, thus contributing to endothelial damage [2].

During pregnancy, the placenta is the primary endocrine organ for maintaining pregnancy and fetal growth. The placenta releases hormones, including androgens, estrogens, and progestogens, and these hormones occur at extremely high concentrations in the maternal circulation. These hormones are important paracrine and autocrine regulators that affect the growth and differentiation of the placental trophoblast, growth and maturation of the placental vascular tree, and uterine endovascular invasion by the extravillous cytotrophoblast [3, 4].

Progesterone and estrogens also function as modulators of uterine vessels in that they decrease the resistance of the spiral uterine arteries and modulate the synthesis and release of angiogenic factors by placental cells. Androgens have the opposite effect. Previous studies have compared the serum levels of sex steroid hormones and signaling in women who had preeclampsia with women who had uncomplicated pregnancies [4–7]. Unsurprisingly, women with preeclampsia exhibited altered serum concentrations of sex steroid hormones. However, no studies have yet examined the role of placental steroid hormone receptors and cord blood steroid hormone concentrations in the pathogenesis of preeclampsia.

The main purpose of the present study is to compare pregnant women with preeclampsia and women with uncomplicated pregnancies in terms of the serum and cord blood levels of sex steroid hormones (estradiol [E2], progesterone [P4], dehydroepiandrosterone sulfate [DHEAS], and testosterone [T]) and placental steroid receptors during the third trimester.

## Materials and methods

### Study design

**Fig. 1:**
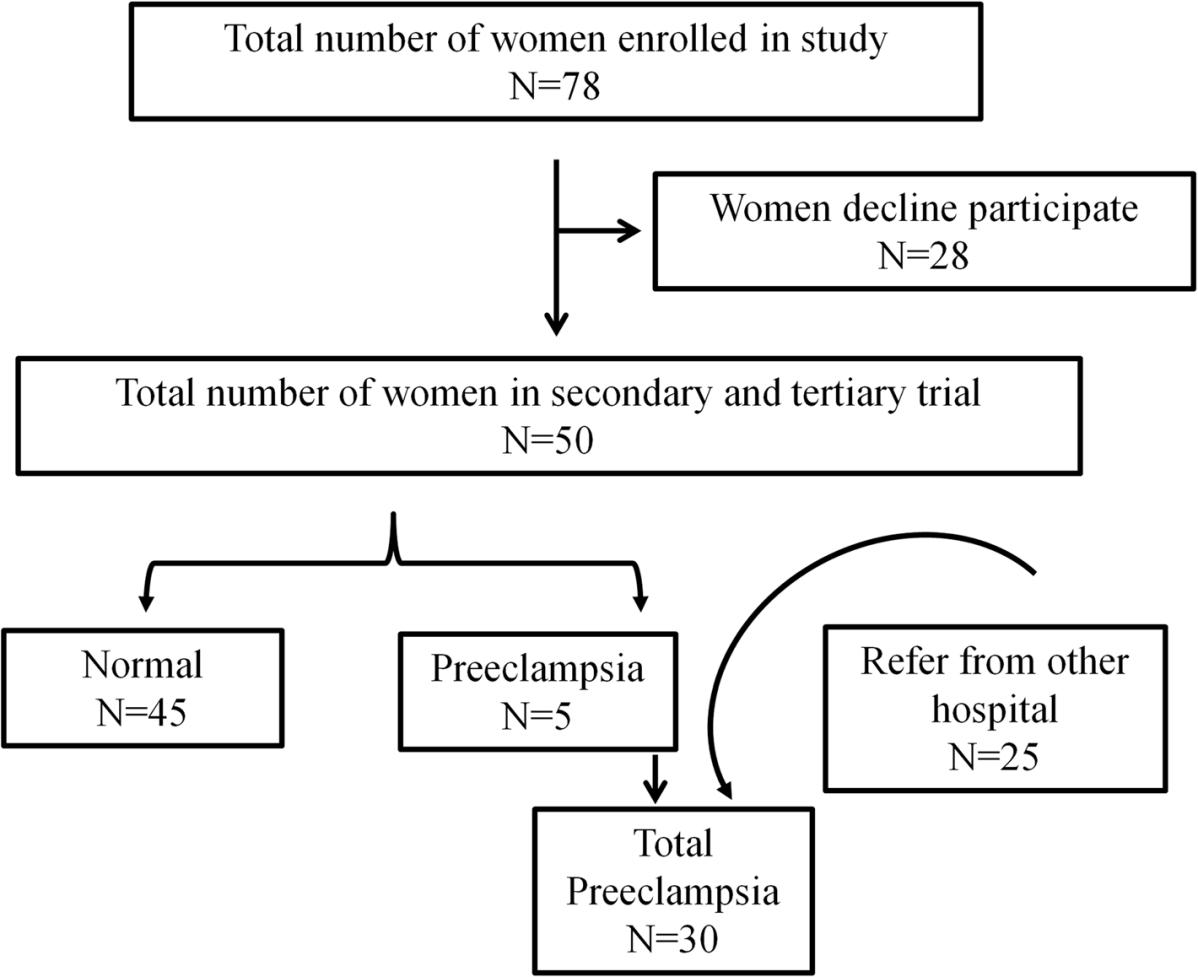
Flow chart of the study population

Following approval of the Ethics Committee of the Institutional Review Board of Chang Gung Memorial Hospital (CGMH 201601484A3) and obtainment of informed written consent from all subjects, this prospective study was conducted from June 2017 to January 2019. Singleton pregnant women (*n* = 78) were recruited to donate blood samples during the first (gestational age: 8–14 weeks), second (gestational age: 20–24 weeks), and third (gestational age: 32–38 weeks) trimesters of pregnancy and to provide cord blood and placenta samples during delivery (Fig. 1). Among the 50 participants who donated 3 serum samples, 45 women had uncomplicated pregnancies and 5 women (10%) developed preeclampsia. During the study period, 25 women with preeclampsia during the second or third trimesters were referred from other hospitals to the high-risk pregnancy prenatal care center of our institute. Thus, there were 45 women in the uncomplicated pregnancy group and 30 women in the preeclampsia group.

Women in the uncomplicated pregnancy group had no diagnoses of preeclampsia or hypertension during pregnancy and no hospitalization due to premature delivery or bleeding. Women in the preeclampsia group were diagnosed based on the presence of new-onset hypertension (systolic blood pressure of 140 mmHg or more, diastolic blood pressure of 90 mmHg or more, or both) on two occasions 6 h or more apart after 20 weeks of gestation and the presence of significant proteinuria (≥300 mg/24 h). None of the patients had a previous history of any known endocrine disorder. Women were excluded if they were smokers, alcoholics, or had chronic maternal disease (essential hypertension, connective tissue diseases, hyperthyroidism, hypothyroidism, chronic glomerulonephritis, renal failure, and diabetes mellitus) or gestational diabetes.

### Tissue and blood collection

Human placental tissues and blood samples of the healthy group (*n* = 45) and the preeclampsia group (*n* = 30) were collected and immediately stored at − 80 °C. Blood was collected in plastic tubes under aseptic conditions, with EDTA as an anticoagulant, and centrifuged at 18,472×*g* for 10 min at 4 °C to separate the serum. Serum concentrations of E2, P4, testosterone and dehydroepiandrosterone sulfate (DHEA-S) were analyzed using commercially available immunoassay systems (ADVIA Centaur XP; Siemens USA). The intraassay and interassay coefficients of variation were 5.0 and 4.1%, respectively, for E2; 5.2 and 3.5% for P4; and 2.6 and 4.3% for testosterone. The intraassay coefficient of variation was 3.9% for DHEA-S.

### Immunohistochemistry

Tissues were paraffin-embedded and subjected to immunohistochemical staining. For this procedure, 4-μm sections were deparaffinized and rehydrated, rinsed in purified water, and treated with 3% H_2_O_2_ for 15 min at room temperature. After rinsing three times with purified water, they were heated in an autoclave for 1 h with 10 mM citrate buffer and then incubated with primary antibodies against estrogen receptor-α (ERα; EP1 diluted 1:50, Bio SB, CA, USA), ERβ (14C8 diluted 1:100, Abcam, Cambridge, UK), progesterone receptor (PR; NCL-L-PGR-312 diluted 1:50, Leica Biosystems, Benton Lane, UK), and androgen receptor (AR; Clone SP107 diluted 1:50, ZECA, CA, USA). After the addition of the appropriate secondary IgG antibody, sections were incubated with DAB (K5007, Dako, Denmark) and counterstained with hematoxylin and eosin (H&E; 1.05174, Merck, MA, USA). Finally, sections were dehydrated in a graded series of ethanol, cleared with xylene, mounted using Histomount (008030, Life Technologies, MD, USA), and coverslips were applied for evaluation by light microscopy.

### Western blot analysis

Placental tissues were washed in PBS and lysed in RIPA lysis buffer (20–188, Merck, MA, USA). Proteins were separated by electrophoresis using 8% SDS/PAGE and then transferred to PVDF Blotting Membranes (10,600,022, GE Healthcare, Germany). Blots were probed with a primary antibody and then developed using Immobilon™ Western (WBKLS0500, Millipore, MA, USA). The primary antibodies were against ERα (MA1–39540, Thermo Fisher, IL, USA), ERβ (PA1-310B, Thermo Fisher, IL, USA), PR (MA1–411, Thermo Fisher, IL, USA), AR (06–680, Millipore, CA, USA), and GAPDH (MAB374, Millipore, CA, USA).

### RNA extraction and quantitative real-time reverse transcription PCR

Total RNA was isolated from placental tissues using the RNA Clean & Concentrator-5 kit (R1014, Zymo Research, CA, USA) and reverse transcribed. Quantitative real-time reverse transcription-PCR (qRT-PCR) was performed using Fast SYBR® Green Master Mix (Applied Biosystems, CA, USA) and the ABI 7500 Fast Real-Time PCR System (Applied Biosystems), with the primers listed in Table 1.

**Table 1:** Primer sequences

	Forward	Reverse
18S	GTAACCCGTTGAACCCCATT	CCATCCAATCGGTAGTAGTG
ER-α	CAGGAACCAGGGAAAATGTG	AACCGAGATGATGTAGCCAGC
ER-β	ACTTGCTGAACGCCGTGACC	CAGATGTTCCATGCCCTTGTT
PR	TGAATCCGGCCTCAGGTAGTT	CGCGCTCTACCCTGCACTC
AR	TCACCGCACCTGATGTGTG	ACATGGTCCCTGGCAGTCTC

### Statistical analysis

SPSS version 10.0 (SPSS, Inc., Chicago, IL, USA) was used for data analysis. Continuous data are summarized as the mean ± standard deviation. Visual inspection and the Shapiro-Wilk normality test were used to check for normality of distributions. The Mann-Whitney rank-sum test was used for the comparison of means. Categorical variables, reported as proportions, were compared using the chi-square test or Fisher’s exact test, as appropriate, and a *P* value below 0.05 was considered statistically significant.

## Results

The preeclampsia group (*n* = 30) and the uncomplicated pregnancy group (*n* = 45) had similar maternal age, parity, gravidity, and hemoglobin levels (Table 2). However, the preeclampsia group had higher blood pressure, maternal body mass index, nulliparity proportion and markedly decreased infant birth weight and gestational weeks.

**Table 2:** Patient characteristics

	Normal pregnant women (*N* = 45)	Preeclampsia women (*N* = 30)	*p*
Age (yr)	33.6 ± 4.7 (24–45)	35.3 ± 5.1 (22–44)	NS
Hemoglobin (g/dL)	11.6 ± 1.53	12.17 ± 1.48	NS
Gestational week (wk.)	38.6 ± 2.8	35.8 ± 2.2	< 0.001
Gravidity	2.1 ± 1.1 (1–5)	2.5 ± 2.2 (1–9)	NS
Parity	1.7 ± 0.7 (1–3)	1.6 ± 0.9 (1–4)	NS
Nulliparity	18 (40%)	19(63.3%)	0.048
Multiparity	27(60%)	11 (36.7%)	0.048
Body Mass Index (BMI)	27.8 ± 4.9	32.3 ± 6.3	0.002
Birth weight (g)	3046.0 ± 717.33	2273.6 ± 886.3	< 0.001
Systolic blood pressure (mmHg)	118.9 ± 23.7	163.0 ± 18.7	< 0.001
Diastolic blood pressure (mmHg)	74.2 ± 12.0	101.8 ± 12.4	< 0.001

*NS* not significant

Data are expressed as mean ± standard deviation

**Fig. 2:**
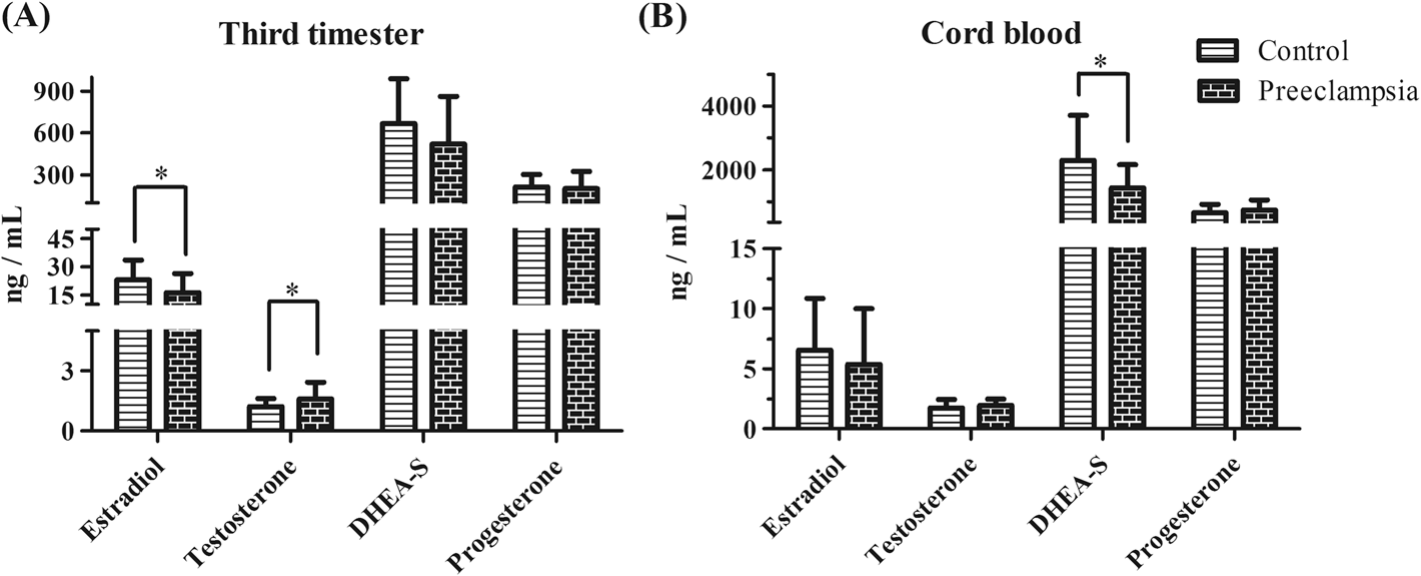
Levels of estradiol, testosterone, DHEAS, and progesterone in women with preeclampsia compared to women with uncomplicated pregnancies. **a** serum in third trimester **b** cord blood. * *P <  0.05*

Comparison of serum data during the third trimester indicated that the uncomplicated pregnancy group had a greater level of serum T and a decreased level of E2, but the two groups had similar levels of P4 and DHEAS (Fig. 2a). Comparison of cord blood indicated that the preeclampsia group had a decreased level of DHEAS, but the two groups had similar levels of P4, E2, and T (Fig. 2b).

**Fig. 3:**
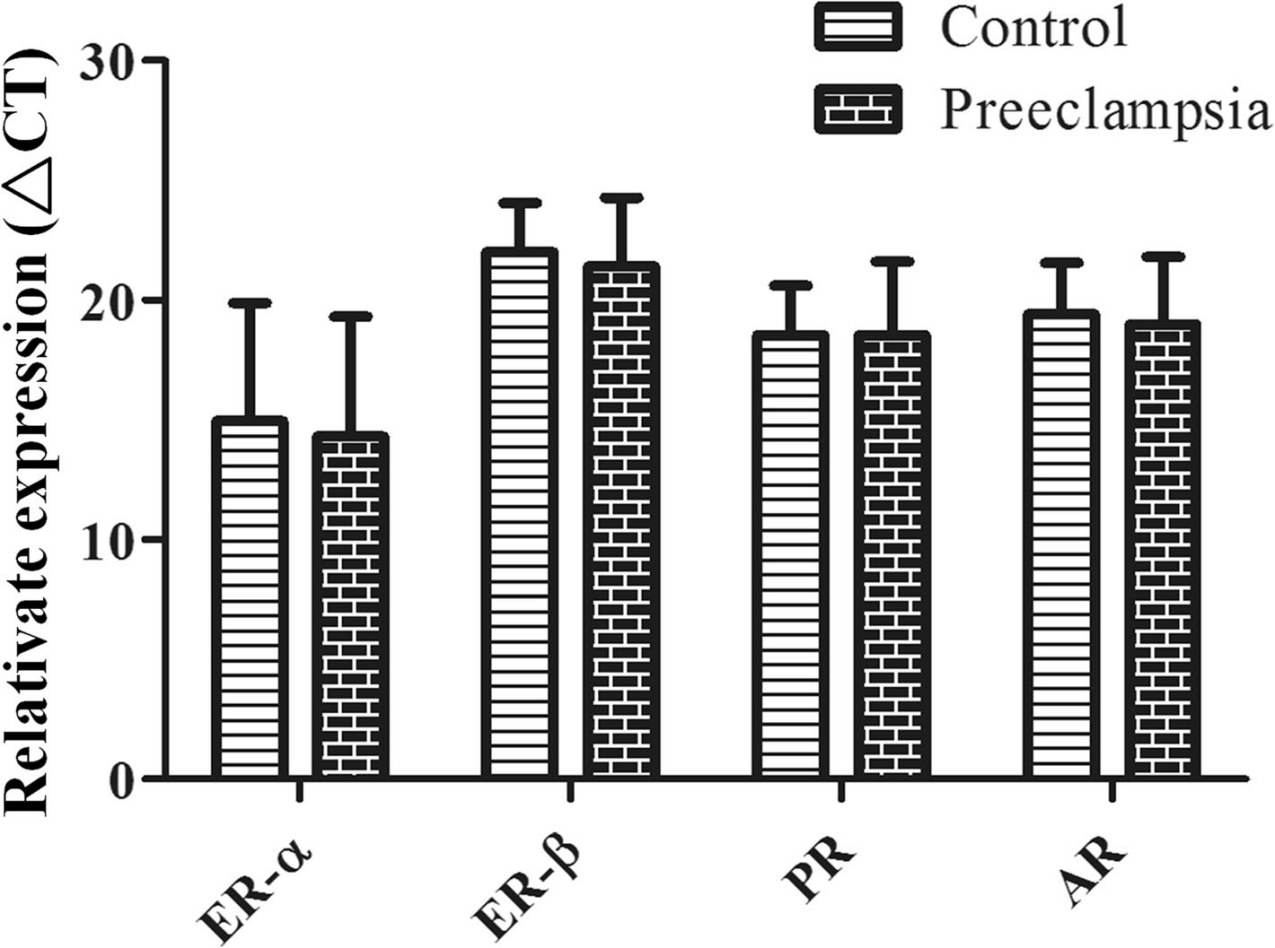
Quantitative evaluation of ERα, ERβ, AR and PR mRNA in the placenta by real-time reverse transcriptase polymerase chain reaction. The two groups had similar mRNA levels of ERα, ERβ, AR, and PR

**Fig. 4:**
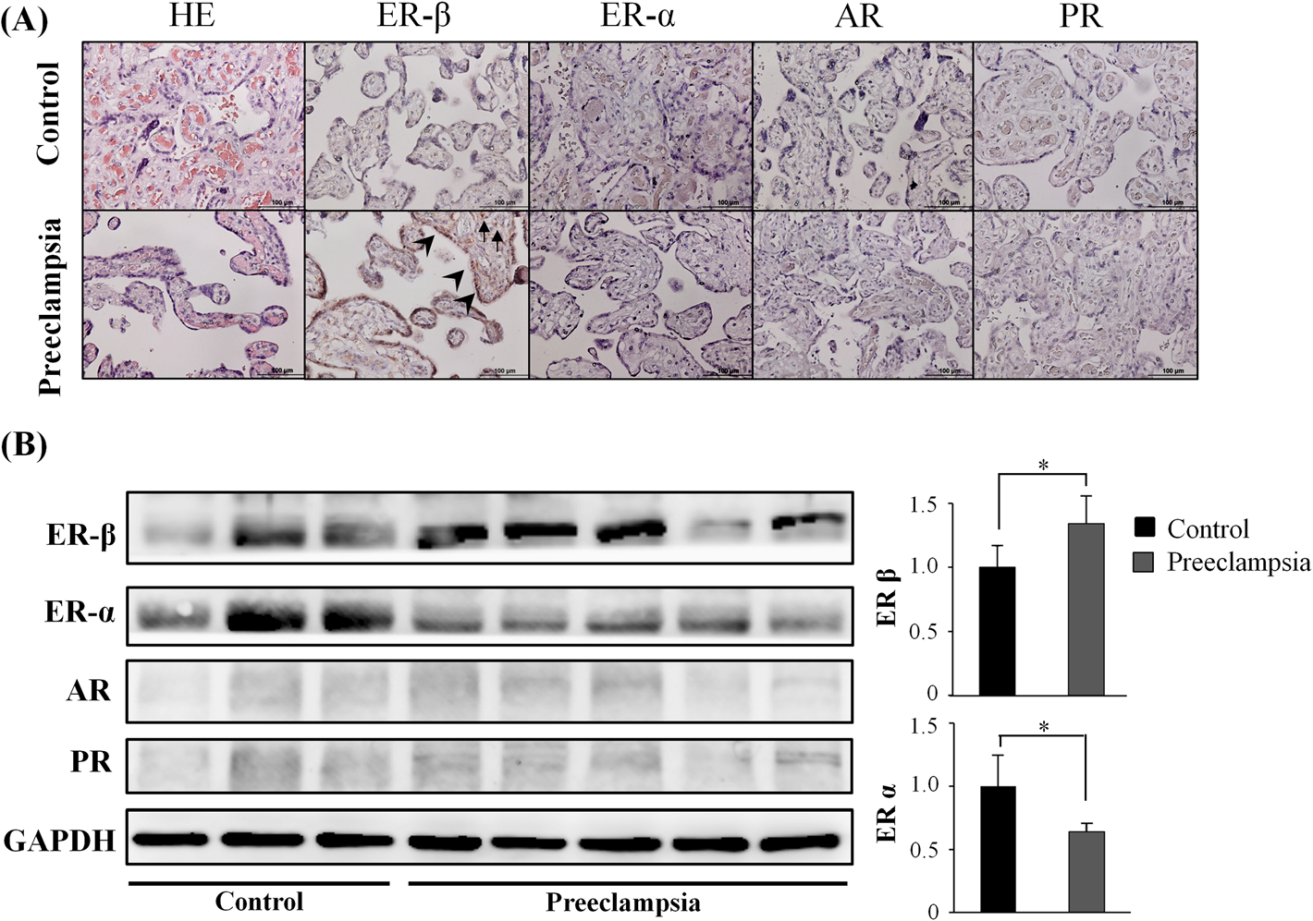
**a** The localization and expression of ERα, ERβ, AR and PR proteins in uncomplicated pregnancies (upper panel) and preeclampsia placenta (bottom panel) were analyzed by immunohistochemical analysis. Specific and robust ERβ immunostaining was detected exclusively in the nucleus and cytoplasm of syncytiotrophoblasts (arrowhead) and stromal cells (arrow). Magnification × 400. **b** Comparison of ERα, ERβ, AR and PR protein expression in placenta from preeclampsia between the two groups by western blot. ERα and ERβ protein expression was significantly lower and higher, respectively, in preeclampsia

We determined the expression of placental hormone receptors in the two groups using western blotting and qRT-PCR. The two groups had similar mRNA levels of ERα, ERβ, AR, and PR (Fig. 3). However, the preeclampsia group had a greater protein level of ERβ and decreased protein level of ERα. The two groups had similar protein levels of AR and PR (Fig. 4b).

We further characterized the expression patterns of these hormone receptors in placental tissues by H&E staining and immunohistochemical analysis of formalin-fixed tissues. The H&E staining results indicated that the cytotrophoblast was surrounded by a layer of syncytiotrophoblasts, a general morphological feature of the placenta. Immunohistochemical staining indicated that ERβ was present in cyto- and syncytiotrophoblast cells of women in the uncomplicated pregnancy group but was mostly present in the nuclei of syncytiotrophoblast cells of women in the preeclampsia group (Fig. 4a). The two groups had no evident differences in the placental distributions of other hormone receptors.

**Fig. 5:**
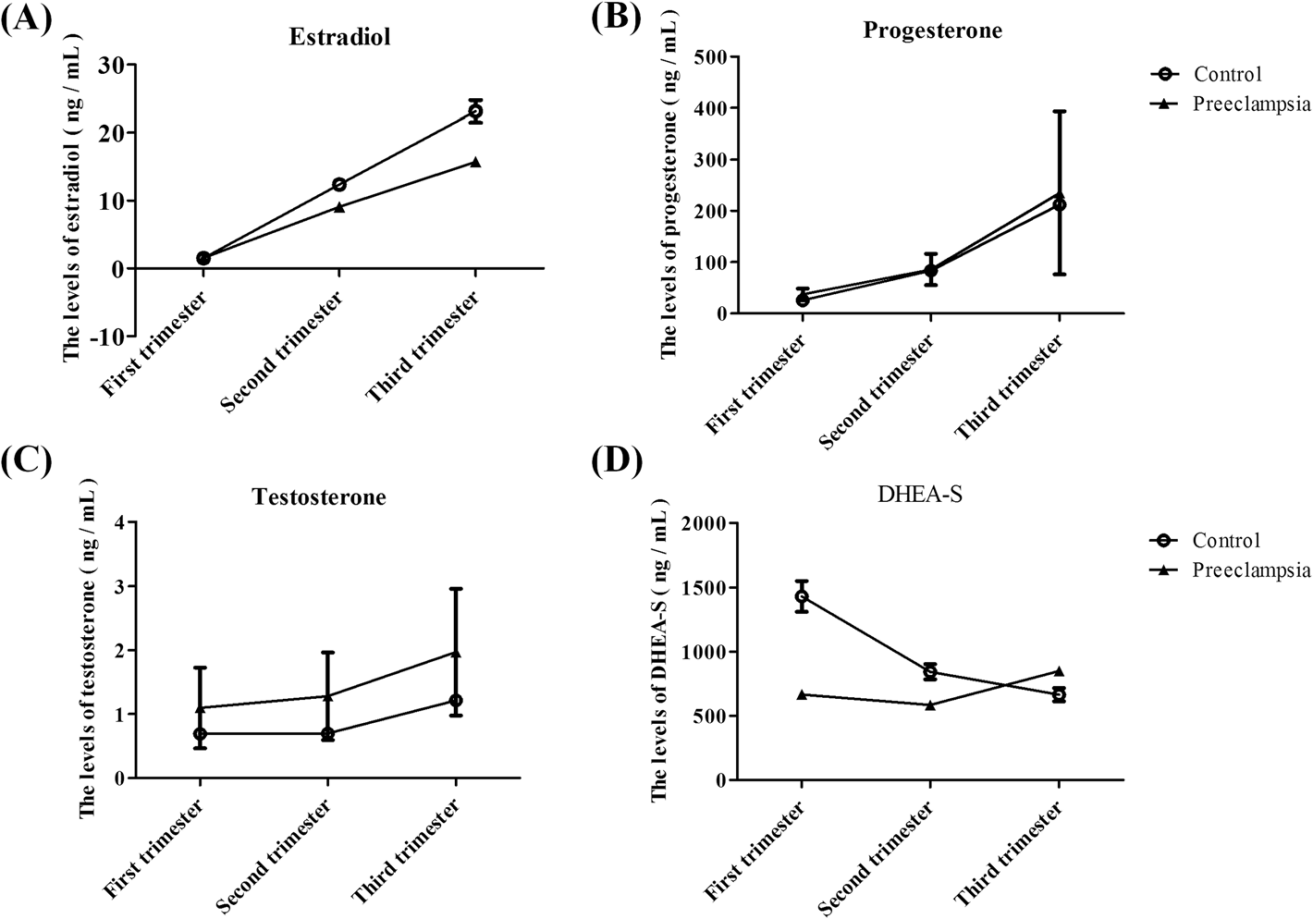
Trajectories of hormone concentrations in women with preeclampsia compared to women with uncomplicated pregnancies. **a** Estradiol **b** Progesterone **c** Testosterone **d** DHEA- S

Measurements of the serum levels of hormones throughout pregnancy indicated that the preeclampsia group had increased T levels but decreased E2 levels (Fig. 5). However, the two groups had similar P4 levels throughout pregnancy.

## Discussion

Female sex steroid hormones, including E2 and P4, have significant physiological roles during menstruation and pregnancy. The ovaries, adrenal glands, and placenta produce these hormones, and the placenta is the major endocrine organ during pregnancy. During pregnancy, the complex process of steroidogenesis occurs in multiple organs, including the maternal uterus, placenta, fetal membrane, and the maternal and fetal hypothalamic-pituitary-adrenal axis, although most steroidogenesis occurs in the placenta.

During pregnancy, the placenta primarily produces estrogen by conversion of androgen precursors that originate in the maternal and fetal adrenal glands. Many studies have reported that serum estrogen levels increase progressively from 22 weeks of gestation and are important regulators of placental and embryo growth [8]. This is consistent with our findings (Fig. 5).

Furthermore, we also found that women in the preeclampsia group had lower serum estrogen levels in the third trimester and lower ERα and higher ERβ protein expression in their placentas. A recent study reported that altered serum concentrations of steroid hormones, including E2, may be associated with preeclampsia [9]. Estrogen has specific metabolic effects in that it stimulates the expression of vascular endothelial growth factor (VEGF) and angiogenesis. However, aberrant production of estrogens could promote preeclampsia because they are exclusively produced by the placenta, and they promote angiogenesis and vasodilation. More recent studies suggested a marked decrease in E2 levels in women with preeclampsia [7]. This is consistent with our findings (Figs. 2a and 5).

Estrogen acts by binding to ERα or ERβ, which are both located in the nucleus but have distinct tissue-specific expression patterns, or by binding to other receptors located in the plasma membrane and cytoplasmic organelles. ERα and ERβ are similar in terms of ligand-binding and nuclear DNA-binding regions, and there are numerous mRNA splice variants for both receptors in diseased and normal tissues. In a ligand-dependent fashion, each ER pool contributes to the overall effects of estrogens on biological outcomes. Kim et al. suggested that placental ERα and ERβ had higher expression at term period compared with early preterm and that they were located in cyto- and syncytiotrophoblast cells [8]. The placentas of our preeclampsia group had a decreased protein level of ERα and an increased protein level of ERβ but no alterations in the levels of the corresponding mRNAs. Many studies have measured the concentration of estrogen during pregnancy, but only Yin et al. examined ERα expression in the placenta of patients with preeclampsia [10]. Their findings are consistent with our findings, although they did not measure the level of ERβ. P4 reduces vascular resistance by decreasing the sensitivity to angiotensin and increasing the production of endothelial vasodilators, which directly affect muscles [10]. A previous study reported that the serum P4 level was markedly increased in women with preeclampsia compared with healthy pregnant women [6]. These results are inconsistent with our data.

The relationship between androgens and maternal cardiovascular and placental function deserves particular consideration because the serum T level in women with preeclampsia is elevated and correlates with vascular dysfunction [5]. Our finding of elevated serum T in women with preeclampsia supports previous studies, thus suggesting that increased androgens and androgen signaling may contribute to preeclampsia [5, 11, 12].

The adrenal cortex produces large amounts of DHEAS and androstenedione during the fetal period, but this production decreases rapidly after birth. However, the physiological role of these changes is still unclear. DHEA is a precursor to testosterone. Interestingly, our results demonstrated that women in the uncomplicated pregnancy and preeclampsia groups had similar serum levels of DHEAS, but the preeclampsia group had a lower cord blood level of DHEAS. The physiological role of DHEAS requires further study.

Our research provided novel insights into the relationship between sex hormone status and preeclampsia. However, our study was limited by the small number of patients. Thus, confirmation requires examination of a larger cohort, with longitudinal data, using highly reliable technology such as gas chromatography/mass spectrometry [7]. In addition, a high BMI is strongly associated with preeclampsia [13], but there is evidence to suggest that obesity may vary by hormone receptor status and by moderating the synthesis and metabolism of circulating sex steroid hormones and related binding proteins [14]. Furthermore, the serum levels of sex hormones change as pregnancy increases, as shown in Fig. 5 and other reports [8]. According to Table 2, the two groups included in this study showed significant differences in gestational age and BMI that might interfere with the levels of hormones and/or the expression of their receptors in the placenta. This important fact is another limitation of the study.

## Conclusions

Our findings suggest that preeclampsia is associated with a distinct hormonal milieu. In particular, we found that E2 and ERα are downregulated and that T and ERβ are upregulated in preeclamptic pregnancies. This suggests the involvement of these molecules in the pathogenesis of preeclampsia.

## Data Availability

The datasets used and/or analyzed during the current study are available from the corresponding author on reasonable request.

## Abbreviations

DHEA: Dehydroepiandrosterone
DHEAS: Dehydroepiandrosterone sulfate
E2: Estradiol
ERα: Estrogen receptor-α
ERβ: Estrogen receptor-β
P4: Progesterone
qRT-PCR: Quantitative real-time reverse transcription-PCR
T: Testosterone
VEGF: Vascular endothelial growth factor
VEGF: Vascular endothelial growth factor
